# Cerebral microsporidiosis manifesting as progressive multifocal leukoencephalopathy in an HIV-infected individual - a case report

**DOI:** 10.1186/1742-6405-11-20

**Published:** 2014-07-15

**Authors:** Maude Loignon, Louise-Geneviève Labrecque, Céline Bard, Yves Robitaille, Emil Toma

**Affiliations:** 1 Département de microbiologie, Infectiologie, immunologie-Université de Montréal, Montréal, Canada; 2 Département de microbiologie et infectiologie, Centre hospitalier de l’université de Montréal (CHUM), Montréal, Canada; 3 Déparetment de radiologie et médicine nucléaire-CHUM, Montréal, Canada; 4 Département de pathologie, Hôpital Sainte-Justine, Montréal, Québec, Canada

**Keywords:** Microsporidiosis, Cerebral lesions, HIV, Progressive multifocal leukoencephalopathy

## Abstract

Microsporidia have become increasingly recognized as opportunistic pathogens since the genesis of the AIDS epidemic. The incidence of microsporidiosis has decreased with the advent of combination antiretroviral therapy but it is frequently reported in non-HIV immunosuppressed patients and as a latent infection in immunocompetent individuals. Herein, we describe an HIV-infected male (46 years) with suspected progressive multifocal leukoencephalopathy that has not responded to optimal antiretroviral therapy, steroids, or cidofovir. Post-mortem examination revealed cerebral microsporidiosis. No diagnostic clue however, was found when the patient was alive. This report underscores the need for physicians to consider microsporidiosis (potentially affecting the brain) when no other etiology is established both in HIV, non-HIV immunosuppressed patients and in immunocompetent individuals.

## Case report

A 46-year-old homosexual male presented at the emergency room on February 22, 2002 for a 5-month progressive visual impairment, headache and occurrence of right hemiparesis in the last week.

He was diagnosed with HIV infection in December 1987. His past history was not significant except for varicella in July 1996. At this previous time, his CD4^+^ count was 650 cells/μL (24%) with a CD4^+^/CD8^+^ ratio of 0.37. In July 2000 and November 2001, the CD4^+^ cell counts were 420 and 330 cells/μL, respectively. No HIV-1 viral load measurements were available for these dates. Until he was admitted to our hospital, the patient had declined any antiretroviral therapy.

**Figure 1:**
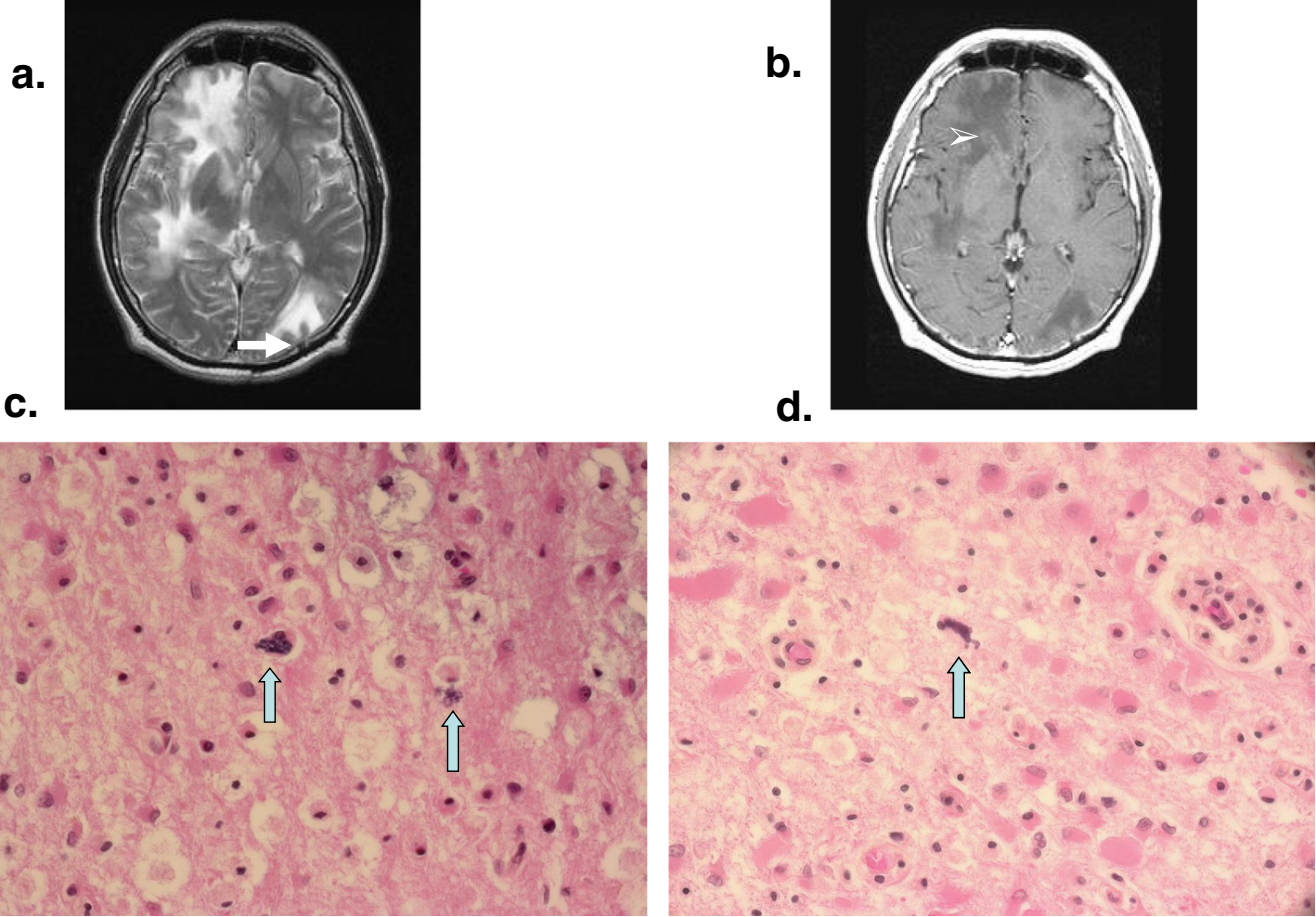
**Brain magnetic resonance imaging (panels a and b) and light microscopy findings on brain specimens from autopsy (panels c and d).** Panel **a**. Axial T2-weighted magnetic resonance image (MRI): multifocal T2 hyperintense subcortical lesions with u-fiber involvement and no mass effect. Panel **b**. T1-weighted MRI with Gadolinium: lesions are hypointense. The frontal lesion shows very little peripheral enhancement. Panel **c**. Brain section-hematoxylin-eosin × 400: white matter showing two clusters of blue, oval shaped microsporidia surrounded by macrophages. Panel **d**. Brain section-hematoxylin-eosin × 400: microsporidia with the characteristic extruded polar tube.

At admission, he was afebrile and his physical examination was unremarkable except for right hemi paresis and left homonymous hemianopsia. His complete blood count (CBC), liver function tests and routine biochemistry were within normal limits. The CD4^+^ count was 340 cells/μL (16%) with a CD4^+^/CD8^+^ ratio of 0.20. A brain CT-Scan and a magnetic resonance imaging (MRI) revealed multifocal coalescent lesions with no mass effect and very little or no enhancement in the white matter of upper left parietal and left occipital, right temporal and frontal lobes with cerebral atrophy, suggestive of progressive multifocal leukoencephalopathy (PML) (Figure 1, panels a and b).

Serologic tests for syphilis (RPR and Treponema/TP-PA), EBV-VCA IgM, toxoplasmosis (IgG and IgM) and search for cryptoccoccal antigen were negative.The patient had positive results for EBV–EBNA-1 IgG, CMV IgG, hepatitis C (anti HCV), hepatitis A, anti HBs, antiHBc. The HBs Ag was negative. He was started on AZT 300/3TC 150 mg (Combivir®) BID and lopinavir 200/ritonavir 50 mg (Kaletra®) 2 tablets BID. The HIV viral load was not performed (for technical reasons) before initiating antiretroviral therapy, but after two weeks it was 2 279 (3.36 log_10_) HIV-1 RNA copies/ml. Because of clinical deterioration, dexamethasone was initiated on March 15, 2002, and continued until April 8, 2002. Since no clinical improvement was apparent, i.v. cidofovir (5 mg/kg) and probenecid twice at one week intervals, then every 2 weeks were started on April 11, 2002 and continued until February 3, 2003. His immune status improved, and on June 10, 2002, the CD4^+^ count showed 440 (26%) with a CD4^+^/CD8^+^ ratio of 0.38, whereas the viral load decreased to 91 HIV-1 RNA copies/mL. Moreover, the patient reported a subjective improvement of his right hemiparesis. On June 26, 2002, AZT was replaced by D4T to avoid anemia due to AZT and probenecid interaction.

The patient was again hospitalized on August 22, 2002 for fever (39°C), seizures and status epilepticus, necessitating admission to the Intensive Care Unit (ICU), for intubation and mechanical ventilation. Anti-convulsive therapy with phenytoin and lamotrigine was then initiated.

Laboratory analysis revealed this time a CD4^+^ count of 380 (21%) with a CD4^+^/CD8^+^ ratio of 0.38 and a HIV viral load below the limit of detection (<50 HIV-1 RNA copies/mL). The brain lesions had not changed since the previous brain MRI. A lumbar puncture was performed on August 23, 2002. The cerebrospinal fluid (CSF) examination revealed proteins 0.53 g/L (normal 0.1-0.5), and glucose 4.3 mmol/L (normal 2.2-3.9). Tests for viral DNA of *Poliomavirus* JC, BK virus, CMV, PCR for herpes group viruses (HSV-1, HSV-2, VZV, CMV, EBV, and HHV-6/7-8), *Mycobacterium tuberculosis*, and search for cryptococcal antigen and VDRL all remained negative in CSF. Plasma CMV DNA, urine cultures, blood cultures for bacteria, *Mycobacteria* and fungi were negative.

An ophthalmologic examination confirmed bilateral blindness of the central origin, but showed no retinitis, keratoconjonctivitis or deep corneal stromal infection. He was discharged on September 19, 2002, and was seen every two weeks at the Ambulatory Unit. On September 23, 2002 his HIV viral load was again below the limit of detection; CD4^+^ count was 350 cells/μL (22%) and CD4^+^/CD8^+^ ratio was 0.36.

Despite an optimal control of HIV infection and continuous combination antiretroviral therapy (cART), the patient’s status did not improve and he was re-admitted to the ICU for status epilepticus on April 28, 2003, intubated and mechanically ventilated. A subsequent brain MRI showed no change as compared with the previous examinations, but was still suggestive of PML. At admission, lactic acid and CK levels, as well as platelet count, remained within normal limits. AST and ALT were slightly elevated: 65 and 67 U/L respectively. While in the ICU, the patient developed multiorgan failure with rhabdomyolysis (CK 47,500), elevated liver enzymes (AST 2 557 U/L), elevated LDH (4 070 U/L) and disseminated intravascular coagulation: thrombocytopenia (12 × 10^9^/L), diminished fibrinogen levels, increased prothrombin time (INR). Lactic acid levels rapidly increased to 17.11 mmol/L on April 29, 2003 before he expired. Rhabdomyolysis and lactic acidosis were probably the consequences of repeated muscular convulsions. Blood and urine cultures remained negative. Search for the cryptococcal antigen was again negative. Permission for post mortem examination was obtained.

At macroscopy, the cerebral hemispheres were unremarkable. The circle of Willis showed a normal architecture without significant arteriosclerotic lesions. Leuko-encephalopathic lesions associated with secondary atrophy predominantly localized in the white matter were noted. These lesions were multifocal and bilateral, the largest observed in the orbito-frontal lobes. Another grey lesion measuring 0.8 × 0.6 cm, which differed from the leuko-encephalopathic lesions was noted in the right internal pallidal nucleus. The corpus callosum had secondary atrophy. Transverse sections of the brain stem showed atrophy of the right bulbar pyramid. Sagittal sections of cerebellum revealed ill-demarcated grey areas within and around dentate nuclei.

At microscopic examination, cerebral microsporidiosis was documented affecting predominantly the white matter (mostly on the right side) of the orbito-frontal lobes with central, right temporo-parietal and cerebellum extension and a right internal pallidal abscess. Intracellular clusters of microsporidial spores were found, some of them with tubular extensions (Figure 1 panels c and d). In addition, a generalized severe anoxic ischemic encephalopathy was noted. Numerous microscopic foci of wallerian degeneration of the perivascular white matter, predominantly fronto-temporo-parietal, compatible with cerebral arterial ischemia were evidenced. No suggestive criteria for PML, such as oligodendrocytic inclusions (*Papova* type) or reactive dysmorphic gliosis were noted; the immunoreactivity for simian virus (SV) 40 was absent. Furthermore, the absence of giant multinucleated cells rendered a diagnosis of HIV encephalopathy improbable. Nonetheless, several extensively calcified small arteries as noted in HIV encephalopathy were present. No immune reactivity to anti Toxoplasma antibodies was present. The Grocott and PAS stainings were negative. The PCR for JCV on brain tissue was not performed.

## Discussion

Microsporidia are widely recognised pathogens in both invertebrates and vertebrates [1]. Microsporidia belong to the phylum *Microsporidia*, with more than 144 genera and 1200 species [1,2]. The most common human pathogens are: *Encephalitozoon*, *Enterocytozoon*, *Pleistophora and Nosema*.

Microsporidia are small (1.5-2.5 μm × 2.5-4 μm), oval shaped, obligate intracellular microorganisms found in epithelial and endothelial cells, fibroblasts, macrophages, astrocytes [1,2]. Although their human pathogenic potential has been reported, they have become increasingly recognized as opportunistic pathogens with the advent of the AIDS epidemic [2]. Initially, *Enterocytozoon bieneusi* was identified as a cause of diarrhoea and *Encephalitozoon* (*E*.) *intestinalis* (*formerly Septata intestinalis*), *and E. cuniculi* for diarrhoeal or disseminated illnesses [2,3].

Microsporidial hepatitis, sclerosing cholangitis, peritonitis, cardiac, sinusal, urinary, pulmonary, renal or ocular involvement have been reported [3]. Microsporidia have been detected in clinical samples from intestines, livers, muscles, corneas, kidneys, adrenals, gonads, ganglia, small arteries, biliary tracts, urine, sinuses, and brain [4,5]. Whereas the incidence of microsporidiosis has decreased in HIV-infected people since the availability of cART, this infection has been increasingly reported in non-HIV-infected individuals, such as solid organ and bone marrow transplant recipients, as well as in cancer, diabetic and elderly patients [6]. Furthermore, microsporidiosis has even been reported in immunocompetent persons [6,7] and in solid organ transplant recipients of latently infected donors [8]. We decided to report this case 12 years later because of this new emerging evidence and increased interest. Moreover, we now seek to alert physicians to potentially include microsporidiosis in the differential diagnosis not only in HIV-infected patients.

Cerebral microsporidiosis was first reported in 1959 [cited by reference [5] and 12 cases due to *E. cuniculi*, all in HIV-infected persons, can be found in the medical literature from 1991 to 1998 [9]. Several other cases were described in immunosuppressed, transplant recipients and HIV-infected individuals [6,10]. In addition, one case was reported in an immunocompetent patient, displaying hemiparesis and epilepsy [7]. Some diagnosed patients benefited from treatment with albendazole, which is active against *E. cuniculi*[11].

In this case report, cerebral microsporidiosis was documented post mortem by morphologic examination of brain samples. Interestingly, this diagnosis was not initially considered when the patient was living. The patient presented with no other clinical manifestations such as diarrhea, keratoconjunctivitis, sinusitis, cholangitis, hepatitis, renal injury, which may have suggested a microsporidial infection. In addition, his CD4^+^ count at admission and 2 months prior was greater than 330 cells/μL in the absence of antiretroviral therapy. Moreover, the brain CT-Scan and MRI findings were suggestive of PML [12].

Unfortunately, no tests for microsporidia were performed and no treatment was initiated while the patient was living, thus, there was no logical reason to suspect microsporidiosis. At necropsy, no other techniques, such as tissue culture, monoclonal antibodies staining, PCR amplification of ribosomal RNA or DNA were performed to identify and characterize the implicated microsporidian species.

It is unclear as to how and when the patient acquired this infection. Microsporidiosis can be transmitted by a respiratory route, contaminated water or food, contact with animals (such as dogs and rabbits), birds, invertebrates or by contact with an infected person [2]. In spite of well-preserved CD4^+^ counts and CD4^+^/CD8^+^ ratios, this patient presented with diminished CD16^+^56^+^ cell counts (10–60 cells/μL; normal 130–700) - the main subpopulation of natural killer (NK) cells. This reduced cell count may have, in part, contributed to his illness. Unfortunately, this finding was not considered during his hospitalisations. Although the T-cell mediated responses are the main protective mechanisms against microsporidiosis, the NK cells may contribute to the immune response and control of this infection [13].

There is growing evidence that latent microsporidiosis is common in immunocompetent individuals and could, therefore, be reactivated during immunosuppression, such as in HIV-infected and immunosuppressed persons, the elderly, transplant recipients, as well as in patients with malignancies or diabetes [6,14]. It is, therefore, possible that our patient experienced a reactivation of latent microsporidiosis that he had acquired before becoming HIV-infected. The diminished CD16^+^56^+^ cell counts may likely be responsible, at least in part, for the reactivation.

We would suggest that cerebral microsporidiosis should be considered in the differential diagnosis of brain lesions in HIV-infected as well as in other immunossupressed patients or transplant recipients, particularly when the etiology is unknown. We would suggest that urinary and CSF specimens should be submitted for detection of Microsporidia. A pre-emptive treatment with albendazole may be considered when the brain lesions do not improve despite optimal HIV control, improved immunity and treatment for other suspected brain lesions.

Collectively, given the ubiquitous nature of microsporidia; their multiple routes of transmission; the potential that a latent infection may be reactivated or transmitted through donated organs; and the multitude of clinical manifestations, this infection should be considered in the differential diagnosis, when no definite etiology is established.

### Consent

Written informed consent for autopsy was obtained from his mandatory and friend, the only next of kin to the patient. A copy of the written consent is available for review by the Editor-in-Chief of this journal. At the time of manuscript writing (11 years after patient’s death) we were unable to identify an individual from whom to seek consent for publication. We informed the Ethical Research Committee and a waiver was granted for consent to publish this case report.

## Competing interests

The authors declare that they have no competing interests.

## Authors’ contributions

MA drafted the manuscript and revised all versions. L-GL contributed to the care of the patient and revised all versions of the manuscript. CB contributed to neuroradiologic examinations, provided MRI images and interpretation and revised the manuscript for publication. YR performed the neuropathological examination, documented the cerebral microsporidiosis, provided the brain section images and interpretation, and revised the manuscript. ET was responsible for the primary care of the patient, revised the draft, prepared the figure for publication and was responsible for the final manuscript. All authors have read and approved the final manuscript.
